# Oridonin attenuates TLR4-driven inflammation and autophagy in LPS-stimulated enteric glial cells: an *in vitro* and *in silico* analysis

**DOI:** 10.3389/fncel.2026.1748505

**Published:** 2026-01-28

**Authors:** Dilara Nemutlu Samur, Aybüke Boyacı, Erkan Maytalman

**Affiliations:** 1 Department of Pharmacology, Faculty of Medicine, Alanya Alaaddin Keykubat University, Antalya, Türkiye; 2 Department of Molecular Medicine, Graduate School of Education, Alanya Alaaddin Keykubat University, Antalya, Türkiye

**Keywords:** autophagy, enteric glia, inflammation, lipopolysaccharide, toll-like receptor 4

## Abstract

**Introduction:**

Enteric glial cells (EGCs) are key regulators of gut-brain axis immunity, and their excessive activation contributes to intestinal inflammation and neuroimmune disturbances implicated in early Parkinson’s disease. Oridonin, a diterpenoid compound with known anti-inflammatory and autophagy-modulating properties, has not been extensively studied in peripheral glial models. Here, we investigated the effects of oridonin on TLR4-mediated inflammatory signaling and autophagy responses in LPS-stimulated EGCs, with molecular docking used as a supportive, hypothesis-generating approach.

**Methods:**

Rat-derived EGCs were exposed to LPS (10 μg/mL) to induce glial activation. Cells were treated with oridonin (1–5 μM) with or without the selective TLR4 inhibitor TAK-242. mRNA levels of TLR4, S100B, LC3, and beclin-1 were quantified by RT-qPCR, while caspase-1 and IL-1β protein levels were assessed by ELISA. Molecular docking was performed to explore potential interactions of oridonin and TAK-242 with the TLR4 receptor complex.

**Results:**

LPS significantly increased TLR4 and S100B expression and upregulated the autophagy markers beclin-1 and LC3. Oridonin dose-dependently suppressed LPS-induced upregulation of TLR4 and S100B and attenuated the elevation of autophagy-related transcripts. Docking studies suggested that oridonin and TAK-242 may interact with regulatory regions of the TLR4 complex, including surface-exposed sites on the TLR4-MD-2 ectodomain and distinct sub-pockets within the intracellular TIR domain, with oridonin exhibiting a stronger predicted binding affinity. Although LPS increased TLR4 mRNA, it elicited only a modest increase in caspase-1 levels and no significant change in IL-1β levels, consistent with incomplete inflammasome activation. Whereas TAK-242 alone did not fully suppress IL-1β, combined treatment with oridonin reduced cytokine levels more effectively, suggesting complementary downstream modulation rather than direct receptor-level synergy.

**Discussion:**

Oridonin exerts powerful anti-inflammatory and autophagy-modulating effects in EGCs by inhibiting TLR4-driven signaling, normalizing excessive autophagic responses, and limiting IL-1β output. Its dual capacity to suppress both upstream (TLR4/S100B) and downstream (caspase-1/IL-1β) components of the inflammatory cascade preserves EGC homeostasis under endotoxin stress. These findings highlight oridonin as a potential modulator of peripheral glial inflammation and support further investigation of its therapeutic potential in gut-brain axis disorders.

## 1 Introduction

Maintaining homeostatic balance within the enteric nervous system (ENS) is essential for the integrity of the gut-brain axis. Increasing evidence suggests that chronic enteric neuroinflammation can promote systemic and central neuroinflammation, which accelerates neurodegeneration (El-Hakim et al., 2022). These persistent inflammatory conditions, together with impaired cellular quality control mechanisms, are now recognized as early contributors to neurodegenerative disorders, most notably Parkinson’s disease (PD) (Han et al., 2022). Consistent with the “gut-first” hypothesis of PD, recent studies demonstrate that PD-related pathology can also be detected within the enteric nervous system (ENS) (Chalazonitis and Rao, 2018) and may even originate in the gut before propagating to the brainstem and cortical regions (Klingelhoefer and Reichmann, 2015). Furthermore, gastrointestinal dysfunction is one of the most prevalent and severe non-motor symptoms of PD (Han et al., 2022). Enteric glial cells (EGCs), which contribute to epithelial barrier maintenance, immune regulation, and GI motility, have emerged as key modulators of enteric homeostasis (Yu and Li, 2014). Under inflammatory conditions, EGCs become reactive and contribute to intestinal neuroimmune dysfunction by releasing proinflammatory mediators, including S100B, IL-1β, and other cytokines (Costa et al., 2019). S100B, a calcium-binding protein predominantly produced by glial cells, has been implicated in promoting glial activation and amplifying inflammatory signaling within the ENS (Angelopoulou et al., 2021). Increasing evidence suggests that inflammatory pathways involving Toll-like receptors (TLRs), particularly TLR4, are expressed by EGCs and participate in gut-derived neuroinflammatory processes relevant to PD pathophysiology (Harris et al., 2006). Activation of TLR4 triggers NF-κB signaling and enhances the production of proinflammatory cytokines (Park et al., 2021). Lipopolysaccharide (LPS), an endotoxin found in the outer membrane of gram-negative bacteria, is a potent inducer of inflammation and exerts its effects primarily through Toll-like receptor 4 (TLR4) (Farhana and Khan, 2025). Some PD patients show higher serum LPS levels than controls, consistent with increased gut permeability and dysbiosis (Forsyth et al., 2011). LPS-induced inflammation can potentiate α-synuclein pathology and neuronal vulnerability through oxidative stress and microglial activation (Massaro Cenere et al., 2024). Therefore, LPS-stimulated enteric glial cells provide a relevant *in vitro* model to investigate peripheral inflammatory mechanisms associated with PD. However, the impact of LPS extends beyond the induction of proinflammatory cytokines. It disrupts cellular quality control mechanisms, particularly autophagy (Ye et al., 2020). Autophagy is a fundamental degradative process responsible for clearing damaged organelles and misfolded proteins, which is vital for maintaining glial health under stress (Gómez-Virgilio et al., 2022; Gómez et al., 2021). In terms of PD, in addition to abnormal protein aggregation, dysregulation of autophagy and lysosomal pathways contributes to the accumulation of α-synuclein and plays a critical role in PD pathogenesis (Hou et al., 2020; Minchev et al., 2022).

Given this interplay between inflammation and autophagic failure, pharmacological agents that can simultaneously modulate both pathways represent a promising therapeutic strategy. Oridonin, a diterpenoid compound derived from *Rabdosia rubescens*, has attracted increasing interest due to its broad anti-inflammatory and neuroprotective actions, including its ability to reduce cytokine production, suppress microglial activation, inhibit inflammasome-related caspase-1 and IL-1β signaling, and modulate autophagy pathways (Xu et al., 2019; Zhang et al., 2013; Yan et al., 2020). These mechanisms overlap substantially with inflammatory and stress-response pathways known to be active in enteric glial cells during gut inflammation. Because EGCs respond robustly to LPS stimulation through TLR4-dependent cytokine release and exhibit autophagy-related alterations under inflammatory stress, oridonin represents a biologically relevant candidate for probing how small-molecule modulators influence these pathways in peripheral glial populations. Despite its documented effects in central neuroinflammatory models, the extent to which oridonin regulates TLR4-associated signaling and autophagy specifically in EGCs remains unclear. Addressing this gap is important for understanding how modulators of glial inflammatory responses may contribute to mechanisms underlying alterations in the gut-brain axis implicated in early PD-related pathology.

In this study, we investigated how oridonin modulates key inflammatory and autophagy-related pathways in an LPS-stimulated EGC model, focusing on markers known to participate in gut-associated neuroimmune responses, including TLR4, S100B, caspase-1, IL-1β, LC3, and beclin-1. Because TLR4 represents a major upstream mediator of LPS-induced signaling in both the central and enteric nervous systems, the selective TLR4 antagonist TAK-242 was employed to assess whether oridonin-induced changes were linked to TLR4-associated mechanisms. By integrating inflammatory and autophagy readouts with pharmacological TLR4 inhibition, this study aims to delineate cellular pathways through which EGCs respond to inflammatory stimuli and to identify how oridonin influences these processes. Such mechanistic insight may help clarify how peripheral glial dysfunction contributes to gut-brain axis disturbances implicated in early PD-related pathology.

## 2 Materials and methods

### 2.1 Enteric glial cell culture

The enteric glial cell line (ATCC^®^ CRL-2690^™^) was generously provided by Dr. Luca Antonioli (University of Pisa). EGCs were chosen for their critical role in maintaining the integrity and function of the ENS, as well as for their involvement in neuroinflammatory processes relevant to PD. The cells were maintained in DMEM supplemented with 10% fetal bovine serum (FBS), 2 mM L-glutamine, and 100 U/mL penicillin–streptomycin. Cells were incubated at 37 °C in a humidified atmosphere with 5% CO2. Upon reaching 70–80% confluency, cells were detached using trypsin–EDTA and seeded into T-25 flasks and 96-well plates for further analysis.

### 2.2 MTT assay for cell viability

Cell viability was evaluated using the MTT (3-(4,5-dimethylthiazol-2-yl)-2,5-diphenyl-2H-tetrazolium bromide) assay, following the procedure described by Grela et al. (2018). Briefly, following 24-h treatments with LPS (5 ng/mL - 50 μg/mL) or oridonin (100 nM - 25 μM), the culture medium was removed, and cells were resuspended in phosphate-buffered saline (PBS; 0.01 M, pH 7.4). MTT solution (5 mg/mL in PBS) was added to each well of the 96-well plate containing treated cells. The plates were incubated for 4 h at 37 °C. After incubation, the supernatant was carefully removed, and 100 μL of dimethyl sulfoxide (DMSO) was added to each well to dissolve the formazan crystals formed by metabolically active cells. The absorbance was measured at 570 nm using a microplate reader (Synergy H1, Biotek, United States). Cell viability was calculated as a percentage relative to the untreated control group (considered 100% viability).

### 2.3 Experimental groups and antagonist applications

For the *in vitro* experiments, EGCs were assigned to the treatment groups summarized in Table 1. To elucidate the role of the TLR4 pathway in the protective effects of oridonin against LPS-induced enteric pathology, cells were treated with the selective TLR4 antagonist TAK-242 (1 μM). This antagonist has been shown to exert effective and specific TLR4 blockade without inducing cellular toxicity (Ono et al., 2020). TAK-242 was added to the culture medium 4 h prior to LPS stimulation. LPS (10 μg/mL) was then administered, followed 4 h later by oridonin (1 or 5 μM), and cells were incubated for a total of 24 h after LPS exposure. Cells between passages 12 and 15 were used for all experiments. Each experimental condition was performed in at least three independent biological replicates (*n* = 3). The groups receiving TAK-242 are listed in Table 2.

**Table 1:** Experimental groups.

Group	Treatment	Description
I	Control	Medium without any treatment
II	LPS	LPS (10 μg/mL)
III	Oridonin (1 μM)	Oridonin at 1 μM
IV	Oridonin (5 μM)	Oridonin at 5 μM
V	LPS + Oridonin (1 μM)	LPS (10 μg/mL) + oridonin (1 μM)
VI	LPS + Oridonin (5 μM)	LPS (10 μg/mL) + oridonin (5 μM)

**Table 2:** Experimental groups treated with the TLR4 antagonist TAK-242.

Group	Treatment
I	Control
II	LPS
III	LPS + Oridonin (1 μM) + TAK-242
IV	LPS + Oridonin (5 μM) + TAK-242

### 2.4 Reverse transcription quantitative polymerase chain reaction

Total RNA was extracted from cells using the RNA Isolation Kit (HibriGen, Cat. No. MG-RNA-01-100) following the manufacturer’s protocol. Briefly, cells were lysed, and total RNA was isolated using a spin-column purification method. RNA quality and concentration were assessed spectrophotometrically, and samples with an OD 260/280 ratio of 1.7–2.0 were used for further analysis. For cDNA synthesis, 1 μg of total RNA was reverse transcribed using the OneScript® Plus cDNA synthesis kit (ABM, G236). The reaction mixture contained 4 μL of RT buffer, 1 μL each of reverse transcriptase, dNTP mix, oligo (dT) primers, and random primers, for a total volume of 20 μL. The mixture was incubated at 25 °C for 10 min, then at 50 °C for 15 min, and finally heated to 85 °C for 5 min to terminate the reaction. The synthesized cDNA was stored at −80 °C until use.

The primer sequences for the target genes (*Tlr4, S100b, Lc3, Beclin1*) are shown in Table 3. PCR amplification was performed using a Roche LightCycler 96 system. The reaction conditions were as follows: pre-denaturation at 95 °C for 5 min, followed by 40 cycles of denaturation at 95 °C for 15 s and annealing/extension at 60 °C for 20 s. A melting curve analysis was performed from 60 °C to 95 °C at 0.5 °C per second to verify the specificity of the PCR products. The relative fold change for each gene was calculated using the 2^−ΔΔCT^ method (Pfaffl, 2001) with the reference gene beta-actin (β-act) for normalization.

**Table 3:** Primer sequences used for RT-qPCR analyses.

Gene	Primer sequence
*Tlr4*	F: GGATGATGCCTCTCTTGCAT R: TGATCCATGCATTGGTAGGTAA
*S100b*	F: GAGGAATGAAGGGCCACTGA R: CCCTAGGCACCAGCAGGTC
*Lc3*	F: CATCAACATTCTGACGGAGCG R: GTTGCTTGGCATCAAACACG
*Beclin-1*	F: GCCTCTGAAACTGGACACG R: CCTCTTCCTCCTGGCTCTCT
*β-actin*	F: CGGCAATGAGCGGTTCC R: TGCCACAGGATTCCATACCC

### 2.5 Enzyme-linked immunosorbent assay

The levels of inflammatory cytokines, caspase-1, and IL-1β were quantified in EGCs lysates using enzyme-linked immunosorbent assay (ELISA) kits. Commercially available ELISA kits for rat caspase-1 (BT Lab, E1897Ra) and IL-1β (BT Lab, E0119Ra) were used according to the manufacturer’s instructions. Briefly, cells were washed with PBS, gently trypsinized, and collected into centrifuge tubes. The suspension was centrifuged at 1,000 g for 5 min, and the supernatant was discarded. Cells were washed three times with PBS to remove residual media. Each 1 × 10^6^ cells was resuspended in 150–250 μL of PBS, and the cells were subjected to three freeze–thaw cycles to ensure complete lysis. The lysates were then centrifuged at 1,500 g for 10 min at 2–8 °C, and the supernatant was collected for ELISA analysis. The supernatants were stored at −20 °C until further use. All measurements were performed in triplicate.

### 2.6 Molecular docking analysis

#### 2.6.1 Protein preparation

The protein structures were prepared in two distinct stages to evaluate both extracellular and intracellular binding potentials of the ligands. First, the three-dimensional (3D) structure of TLR4 (PDB ID: 3FXI) was downloaded from the Protein Data Bank (PDB) to represent the extracellular domain. The binding region of the protein was identified, unnecessary water molecules were removed, and only amino acids crucial for the binding site were retained. We used the TLR4-MD-2 complex rather than TLR4 alone because the physiologically relevant ligand-binding pocket is formed by MD-2, not by TLR4 alone. MD-2 is essential for proper ligand accommodation, orientation, and TLR4 activation. Second, to investigate the intracellular signaling domain, the high-confidence predicted structure of human TLR4 was obtained from the AlphaFold Protein Structure Database (UniProt: O00206). To focus specifically on the signaling machinery and eliminate computational interference from the large extracellular portion, the TIR domain (residues 670–839) was isolated by using ChimeraX (Lin et al., 2012). SwissDock employs a blind docking strategy using the AutoDock Vina algorithm, which automatically scans the entire protein surface to predict the most favorable binding pockets based on energy calculations. Therefore, no predefined binding region was specified. The binding sites and clusters generated by SwissDock represent the lowest-energy binding conformations across the entire protein surface.

#### 2.6.2 Ligand preparation

The canonical SMILES of oridonin (PubChem CID: 5321010) and TAK-242 (PubChem CID: 11703255) were obtained from the PubChem^1^ (accessed on 9 October 2024) and uploaded to the SwissDock platform.

#### 2.6.3 Docking protocol

The *Autodock Vina* algorithm, provided by SwissDock, was used to evaluate ligand binding affinities. The grid box dimensions were set to 30 × 30 × 30 Å to encompass the predicted binding pocket. The center of the grid was adjusted depending on the predicted ligand-binding region (Box center: 11.0 0.0–4.0 for TLR4). The spacing between grid points was set to the default value (0.375 Å), and the sampling exhaustivity was set to 20 to ensure reliable pose prediction; 20 docking conformations were generated per protein-ligand pair. All other parameters were set to the default values provided by the SwissDock platform. The binding affinities (kcal/mol) and binding poses were analyzed to identify the most favorable interactions between the ligands and the proteins.

#### 2.6.4 Visualization of results

Output files and three-dimensional binding positions provided by SwissDock were visualized using ChimeraX v1.9 for detailed structural analysis, including key hydrogen bonds and van der Waals interactions identified.

### 2.7 Statistical analysis

Data were expressed as the mean ± standard error of the mean (SEM). Before performing parametric tests, data distributions were tested for normality using the Kolmogorov–Smirnov test. Differences between groups were analyzed using one-way ANOVA followed by Tukey post-hoc test. For data sets that did not follow a normal distribution, the Kruskal-Wallis test was performed, followed by Dunn’s test for *post hoc* comparisons between groups. All statistical analyses were performed using GraphPad Prism 10.0 software (SanDiego, CA, United States). A *p*-value less than 0.05 was considered statistically significant.

## 3 Results

### 3.1 LPS and oridonin exhibit dose-dependent effects on EGC viability

**Figure 1:**
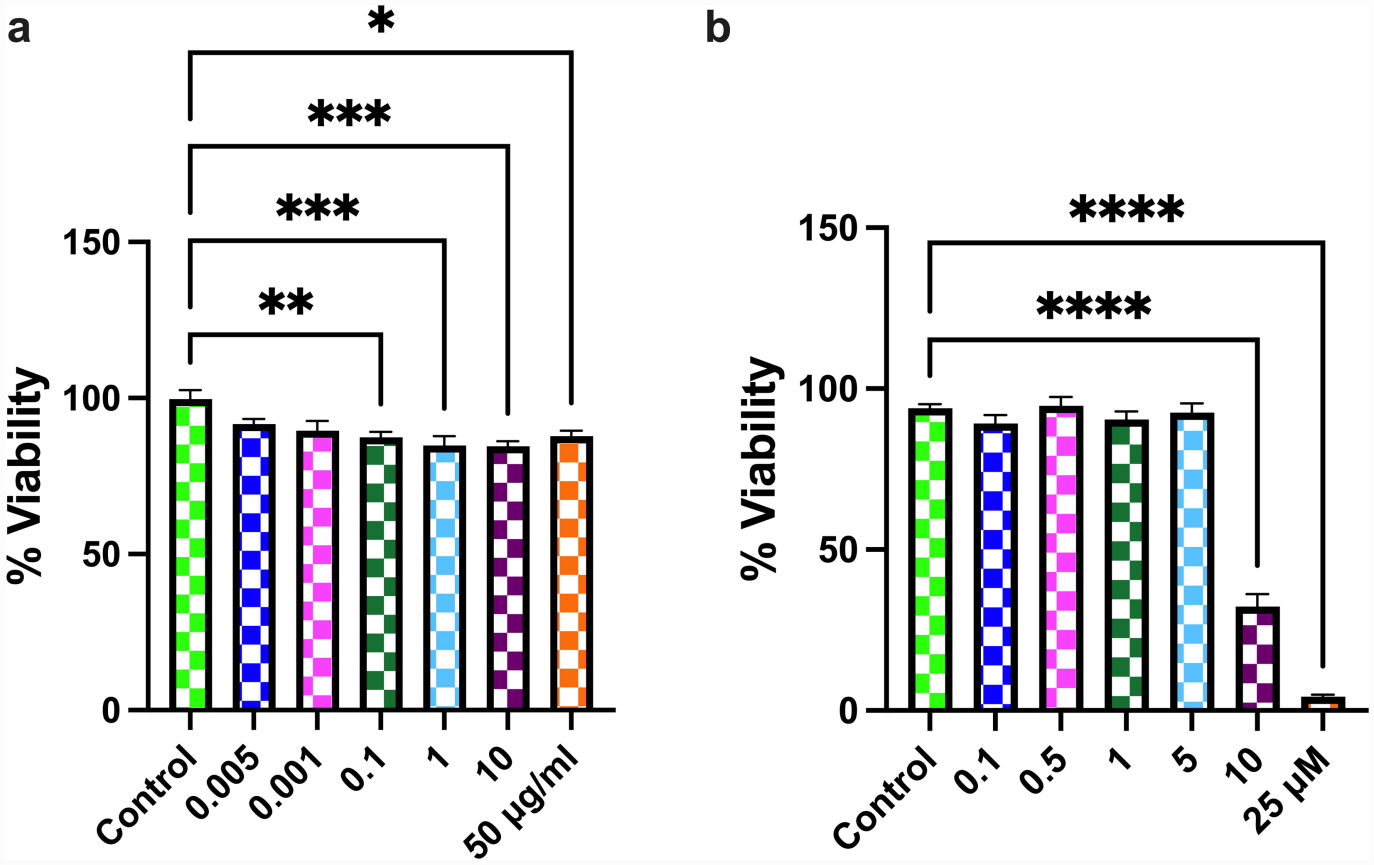
Effects of LPS **(a)** and oridonin **(b)** on cell viability assessed by the MTT assay. Cell viability is expressed as percentage of control (% viability relative to untreated cells). Data represent mean ± SEM obtained from three independent experiments; each performed with eight technical replicates per condition. Statistical analyses were performed using one-way ANOVA followed by Tukey’s *post hoc* test. Data represent the mean values obtained from three independent experiments, each conducted with eight technical replicates. *p < 0.05, **p < 0.01, ***p < 0.001, ****p < 0.0001.

Cell viability analyses were performed to determine the appropriate concentrations of LPS and oridonin for subsequent experiments (Figure 1). Although LPS produced a statistically significant reduction in cell viability at concentrations ≥ 0.1 μg/mL, the decrease remained moderate across the entire dose range, with viability values remaining within approximately 80–90% of control levels (Figure 1a). Notably, the 10 μg/mL concentration maintained cell viability at ~85%, indicating that it elicited measurable cellular stress while remaining well within the sub-cytotoxic range. We aimed to model a subacute, sustained inflammatory challenge relevant to enteric glial activation under conditions of intestinal barrier dysfunction, where luminal LPS exposure can be markedly higher than systemic or CNS levels (Ghosh et al., 2020). The 10 μg/mL dose met this criterion and is also widely used in glial and enteric inflammation models to produce a reliable proinflammatory response (Tawarayama et al., 2023). Therefore, 10 μg/mL LPS was selected as the optimal dose for subsequent experiments. For oridonin, the dose–response curve demonstrated a clear concentration-dependent reduction in cell viability (Figure 1b). While lower concentrations (1 and 5 μM) maintained viability within the 90–93% range, indicating that they remained within a sub-cytotoxic window, a marked drop in viability was observed at 10 μM (mean 32.3%), and viability decreased to below 5% at 25 μM. Because concentrations ≥ 10 μM produced overt cytotoxicity that could confound the interpretation of inflammatory and autophagy-related mechanisms, only non-cytotoxic concentrations were selected for subsequent experiments. Thus, 1 μM and 5 μM were chosen as the low and high concentrations of oridonin, respectively, as they reliably preserved metabolic activity while allowing assessment of oridonin’s modulatory effects under inflammatory conditions.

### 3.2 Oridonin attenuates the LPS-induced upregulation of S100B, TLR4, and beclin-1 mRNA in enteric glial cells

**Figure 2:**
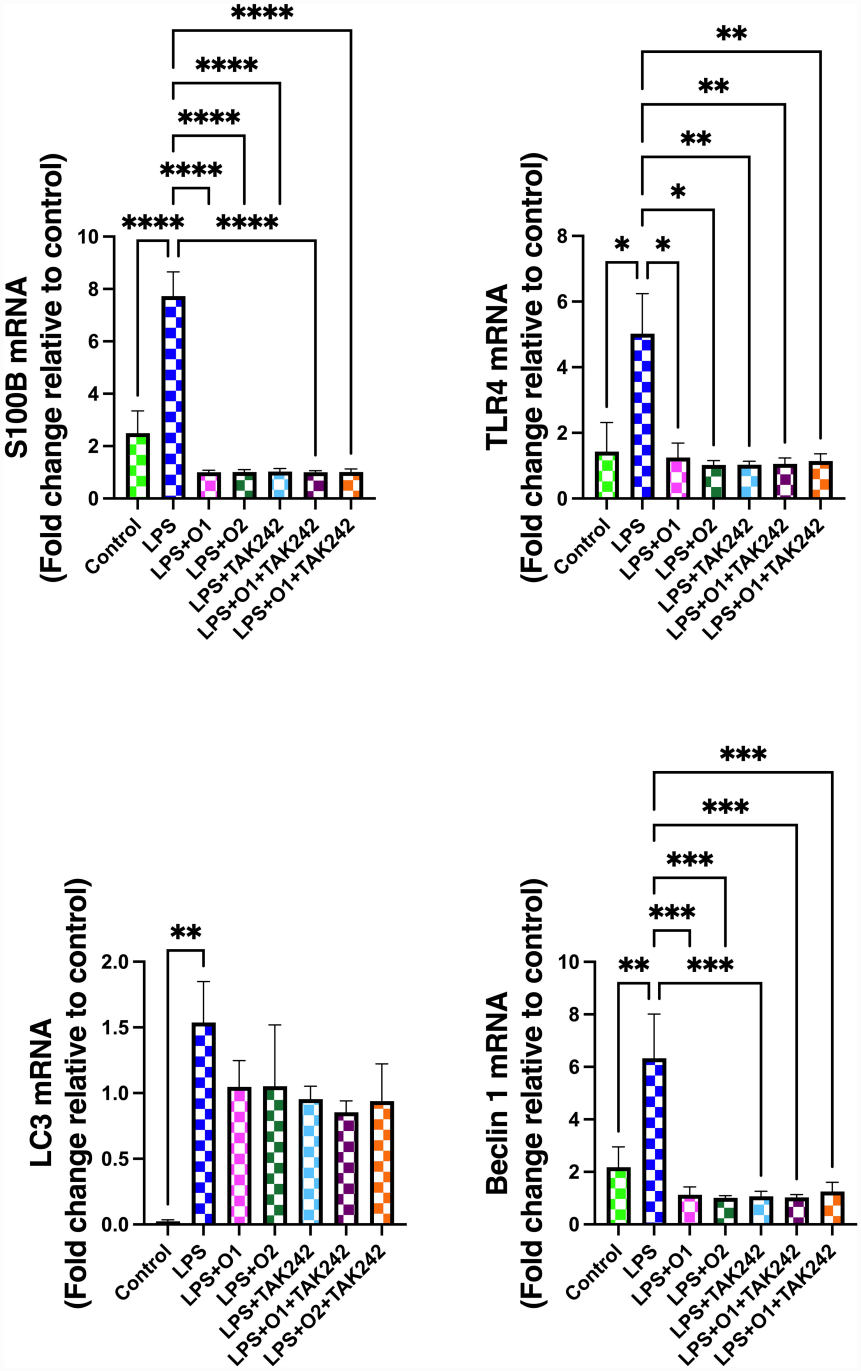
Effects of LPS and oridonin on S100B, TLR4, LC3, and beclin-1 mRNA levels in enteric glial cells. Data represent mean ± SEM from three independent experiments, each performed in technical replicates. Statistical analyses were performed using one-way ANOVA followed by Tukey’s *post hoc* test. *p < 0.05, **p < 0.01, ***p < 0.001, ****p < 0.0001. LPS: ipopolysaccharide; O1: 1 μM oridonin; O2: 5 μM oridonin.

Figure 2 illustrates the changes in S100B, TLR4, LC3, and beclin-1 mRNA levels in EGCs. LPS stimulation induced a significant increase in S100B mRNA expression compared with the control group (*p* < 0.0001). This elevation was significantly reduced by both 1 μM and 5 μM oridonin (*p* < 0.0001 for both). Treatment with the TLR4 antagonist TAK-242 also significantly lowered S100B mRNA expression relative to the LPS group (*p* < 0.0001), although no significant differences were observed when compared with the oridonin-treated groups. Similarly, TLR4 mRNA expression was significantly higher in the LPS group than in the control group (*p* < 0.05). This increase was significantly attenuated by 1 μM and 5 μM oridonin (*p* < 0.05 for both). TAK-242 treatment significantly reduced TLR4 mRNA levels compared with LPS alone (*p* < 0.01), but its effect did not differ significantly from the groups receiving oridonin without antagonist.

LC3 mRNA expression was elevated only in the LPS group when compared with the control (*p* < 0.01). Although oridonin treatment produced a partial reduction in LC3 levels at both concentrations, these changes did not reach statistical significance. Beclin-1 mRNA expression was also significantly increased in the LPS group relative to the control (*p* < 0.01). This increase was significantly reversed by both 1 μM and 5 μM oridonin (*p* < 0.001 for both). TAK-242 treatment resulted in a significant reduction in beclin-1 levels compared with the LPS group (*p* < 0.001), but no significant differences were observed compared with oridonin alone.

### 3.3 Oridonin synergistically modulates caspase-1 and IL-1β levels in combination with TLR4 inhibition

**Figure 3:**
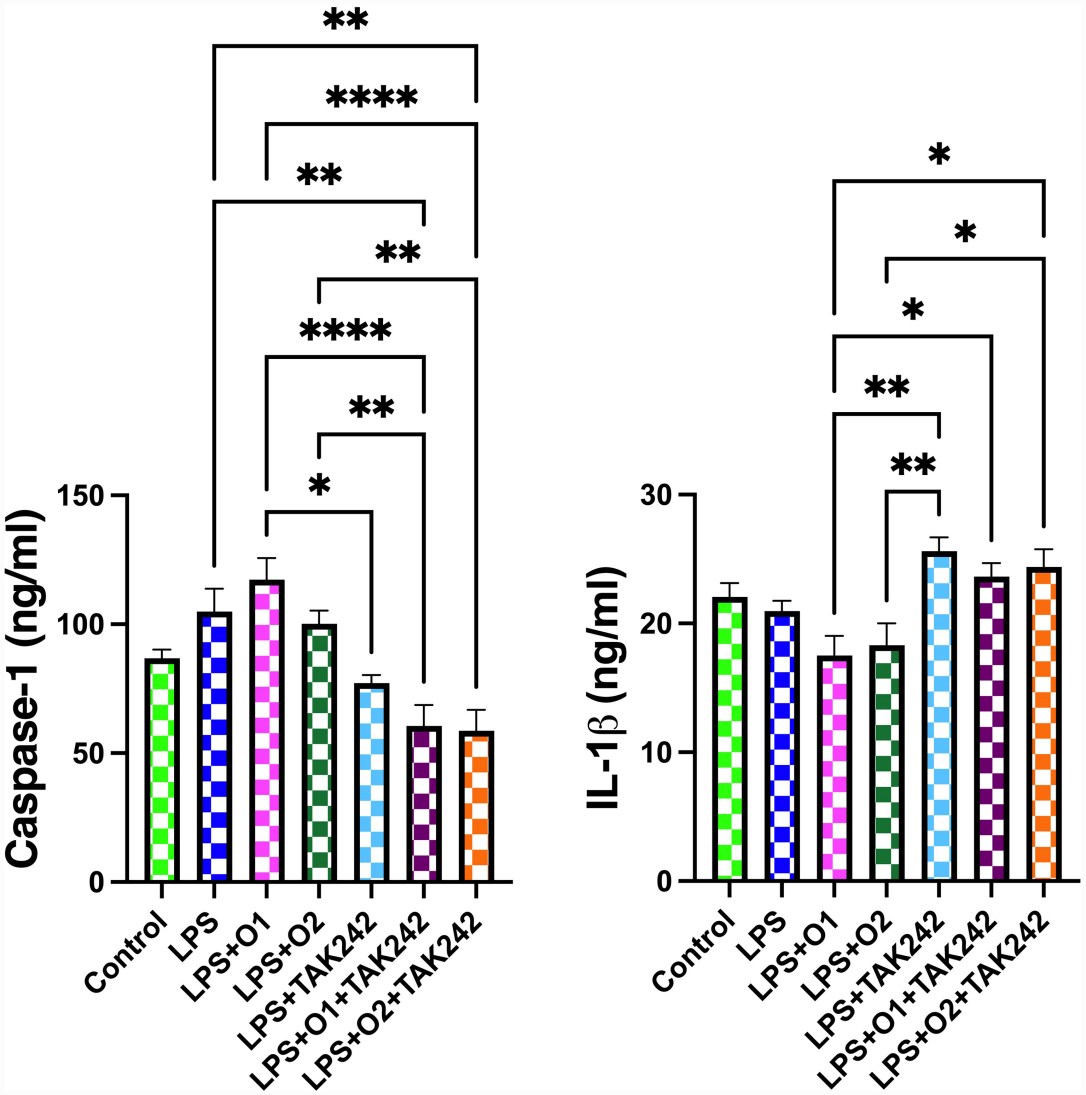
Effects of LPS and oridonin on caspase-1 and IL-1β levels in enteric glial cells. Data represent mean ± SEM from three independent experiments, each performed in technical replicates. Statistical analyses were performed using one-way ANOVA followed by Tukey’s *post hoc* test. *p < 0.05, **p < 0.01, ****p < 0.0001. LPS: Lipopolysaccharide; O1: 1 μM oridonin; O2: 5 μM oridonin.

LPS administration increased caspase-1 levels in EGCs by approximately 21% (Figure 3). Treatment with 1 or 5 μM oridonin did not produce any change in this increase. With the administration of the TLR4 antagonist TAK-242, caspase-1 levels were found to be significantly lower compared with the LPS + O1 and LPS + O2 groups (*p* < 0.0001 and *p* < 0.01, respectively). Although IL-1β levels did not differ significantly between the control and LPS groups, they were significantly higher with TAK-242 administration than in the LPS + O1 and LPS + O2 groups (*p* < 0.05 for both).

### 3.4 Oridonin displays significantly higher predicted binding affinity for the extracellular TLR4-MD2 complex and intracellular TIR domains compared to TAK-242

**Figure 4:**
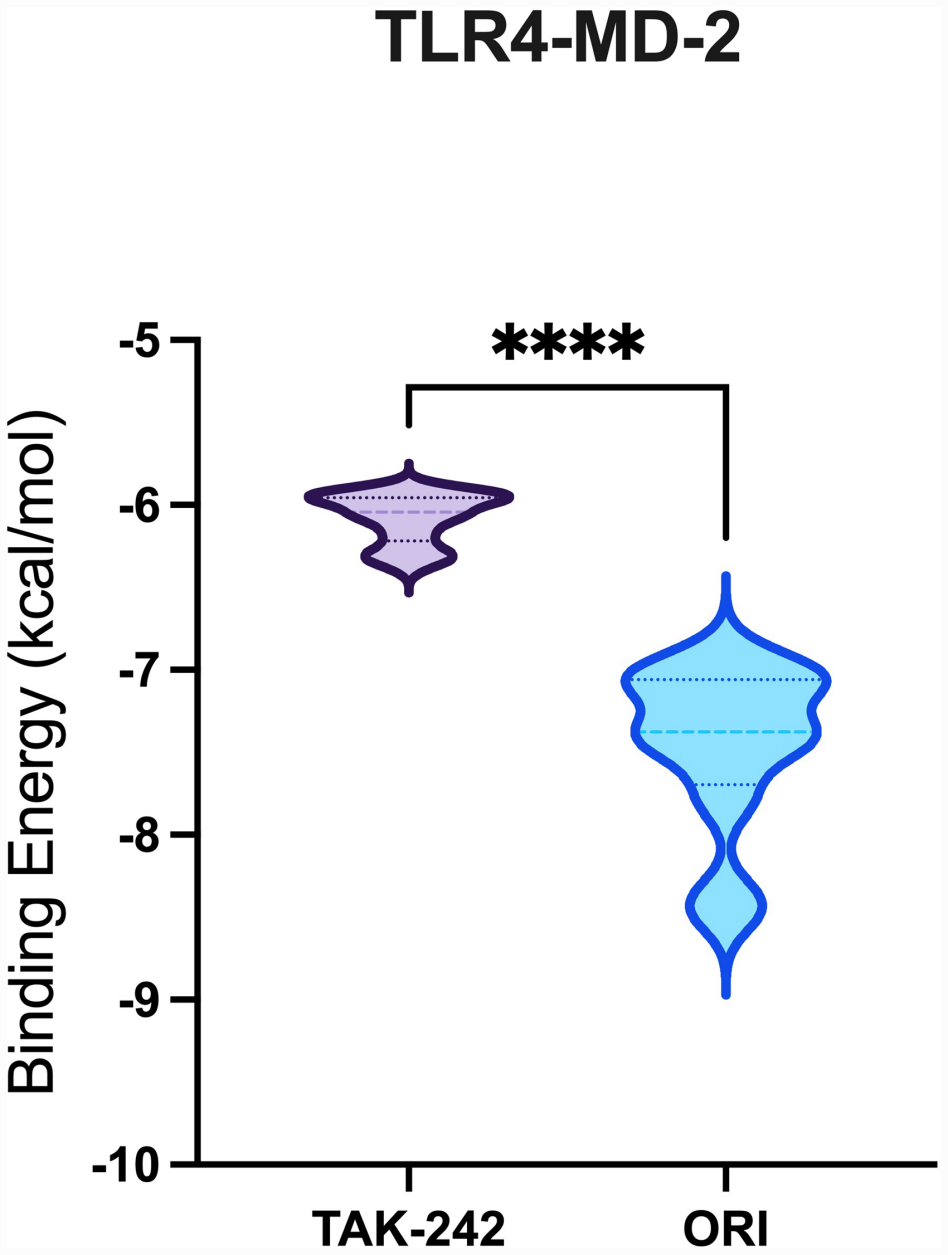
Violin plot showing the distribution of predicted binding affinities (docking scores) of oridonin and TAK-242 against the TLR4-MD2 complex. Each violin depicts the spread and density of docking scores across 20 poses per protein, with wider sections indicating a higher frequency of poses around that binding energy. Lower (more negative) scores represent stronger predicted binding affinity. ****p < 0.0001.

**Figure 5:**
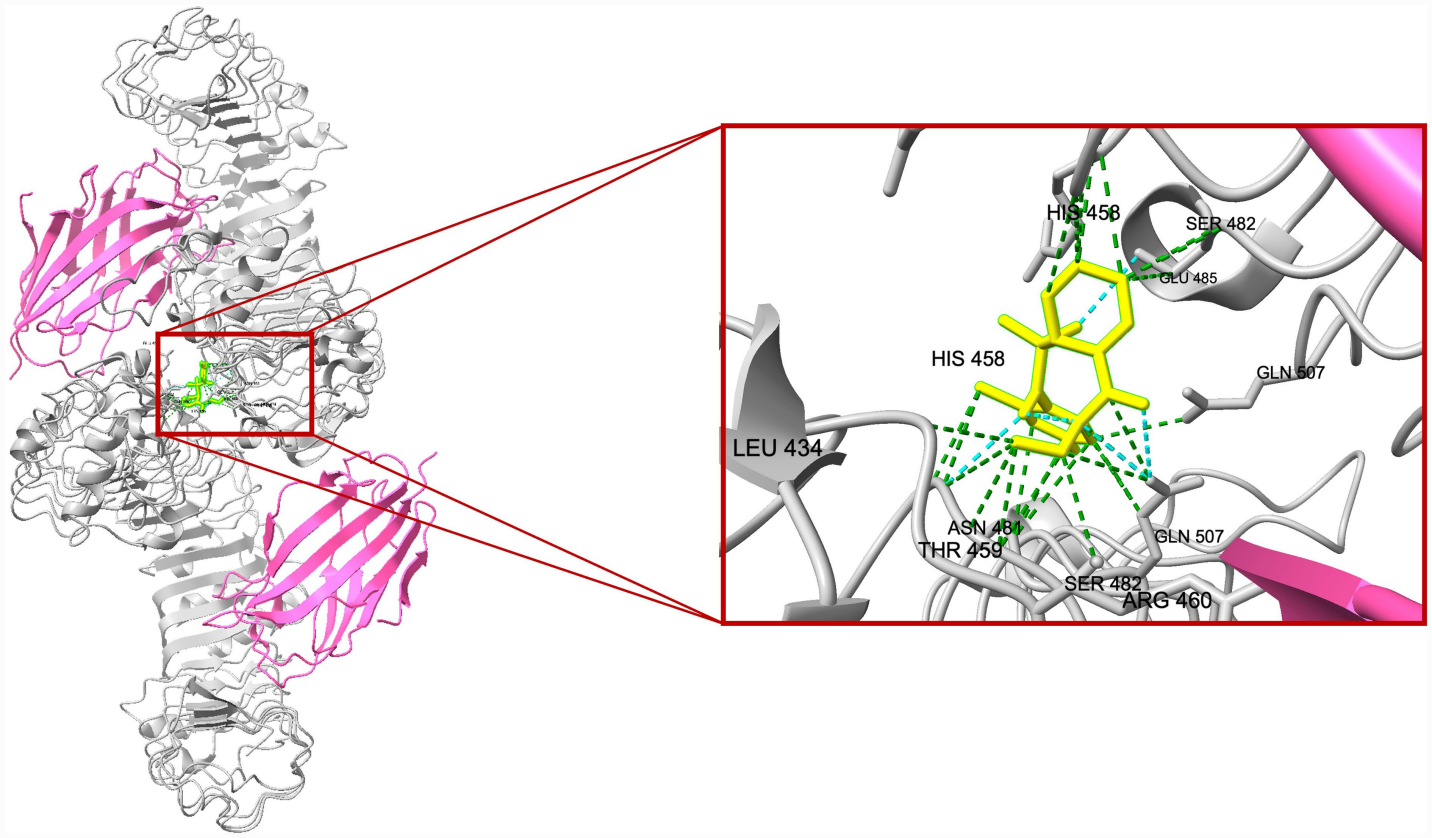
Predicted binding pose of oridonin within the TLR4 extracellular domain. The TLR4-MD-2 complex is shown in cartoon representation (TLR4 in grey, MD-2 in pink). Oridonin is depicted in a yellow stick representation. The zoomed-in panel highlights the interaction network stabilizing oridonin in the identified binding pocket. Key interacting residues (e.g., HIS458, GLY480, SER482, GLN507) are labeled. Green dashed lines indicate predicted van der Waals contacts, while cyan dashed lines represent potential hydrogen bonds.

**Figure 6:**
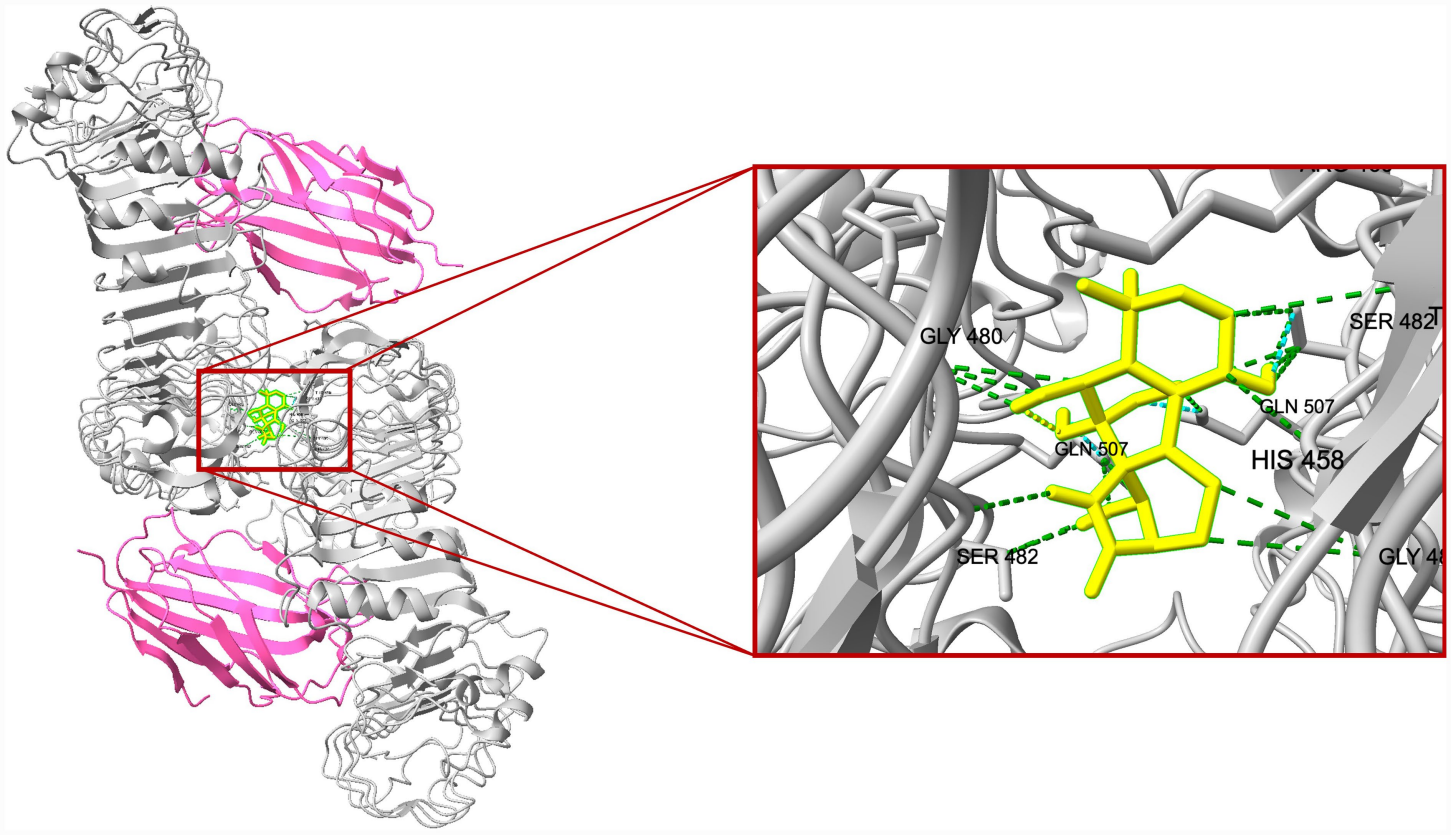
Predicted binding pose of TAK-242 within the TLR4 extracellular domain. The TLR4-MD-2 complex is shown in cartoon representation (TLR4 in grey, MD-2 in pink). TAK242 is depicted in a yellow stick representation. The zoomed-in panel highlights the interaction network stabilizing oridonin in the identified binding pocket. Key interacting residues (e.g., HIS458, THR459, ARG460, GLY480, ASN481, SER482, GLN507) are labeled. Green dashed lines indicate predicted van der Waals contacts, while cyan dashed lines represent potential hydrogen bonds.

**Figure 7:**
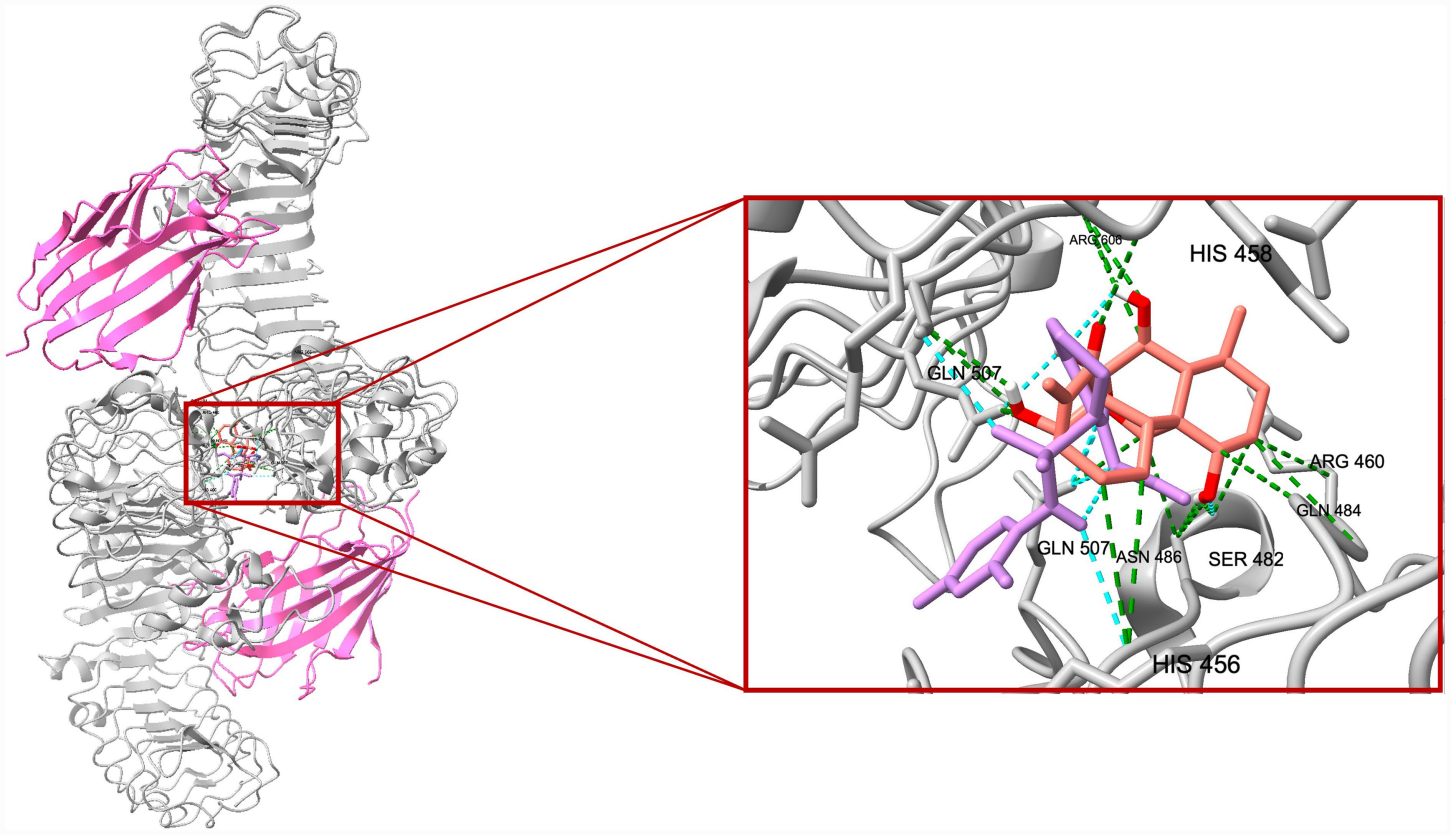
Comparative visualization of the binding modes of oridonin (red) and TAK-242 (purple) within the TLR4-MD-2 complex. Both ligands are shown in stick representation, with the surrounding TLR4 residues displayed in gray and MD-2 in magenta. Green dashed lines represent predicted van der Waals contacts, while cyan dashed lines indicate hydrogen-bond interactions stabilizing each ligand.

**Figure 8:**
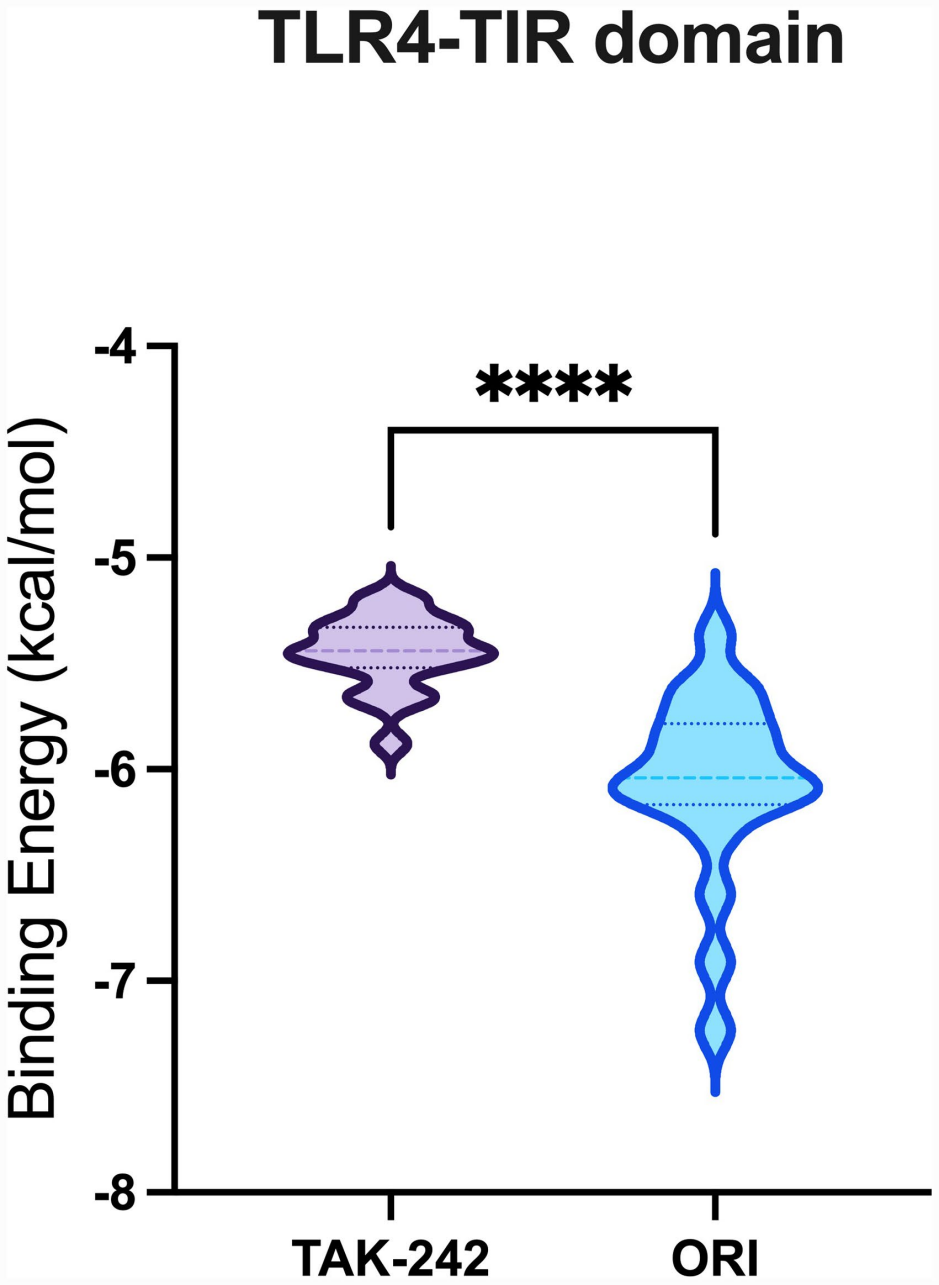
Violin plot showing the distribution of predicted binding affinities (docking scores) of oridonin and TAK-242 against TLR4-TIR domain. Each violin depicts the spread and density of docking scores across 20 poses per protein, with wider sections indicating a higher frequency of poses around that binding energy. Lower (more negative) scores represent stronger predicted binding affinity. ****p < 0.0001.

**Figure 9:**
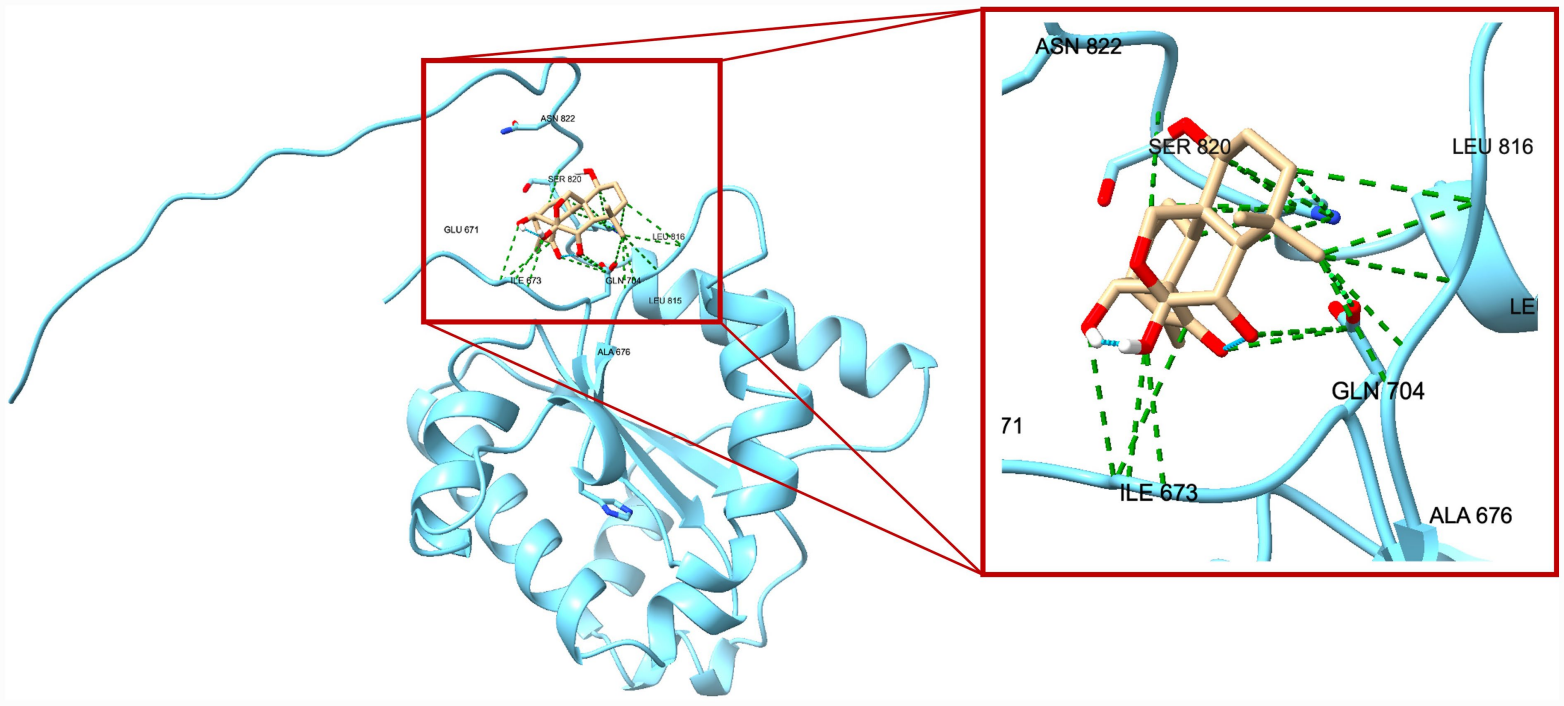
Predicted binding pose of oridonin within the TLR4 intracellular domain. The TLR4-TIR domain is shown in blue. Oridonin is depicted in beige stick representation. The zoomed-in panel highlights the interaction network stabilizing oridonin in the identified binding pocket. Key interacting residues (e.g., ILE673, GLN704, SER820, and ASN822) are labeled. Green dashed lines indicate predicted van der Waals contacts, while cyan dashed lines represent potential hydrogen bonds.

**Figure 10:**
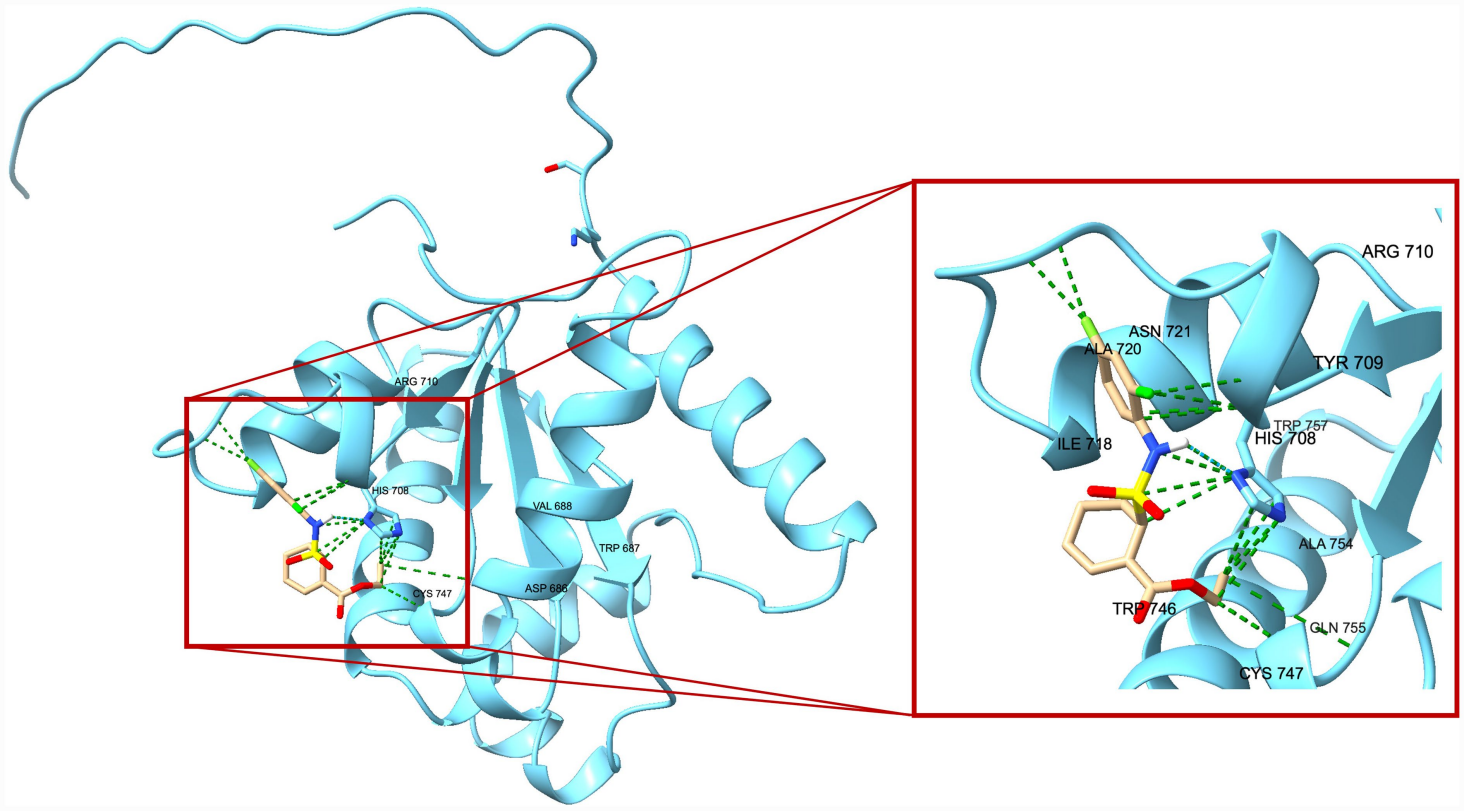
Predicted binding pose of TAK-242 within the TLR4 intracellular domain. The TLR4-TIR domain is shown in blue. TAK-242 is depicted in beige stick representation. The zoomed-in panel highlights the interaction network stabilizing oridonin in the identified binding pocket. Key interacting residues (e.g., HIS708, ILE718, and CYS747) are labeled. Green dashed lines indicate predicted van der Waals contacts, while cyan dashed lines represent potential hydrogen bonds.

**Figure 11:**
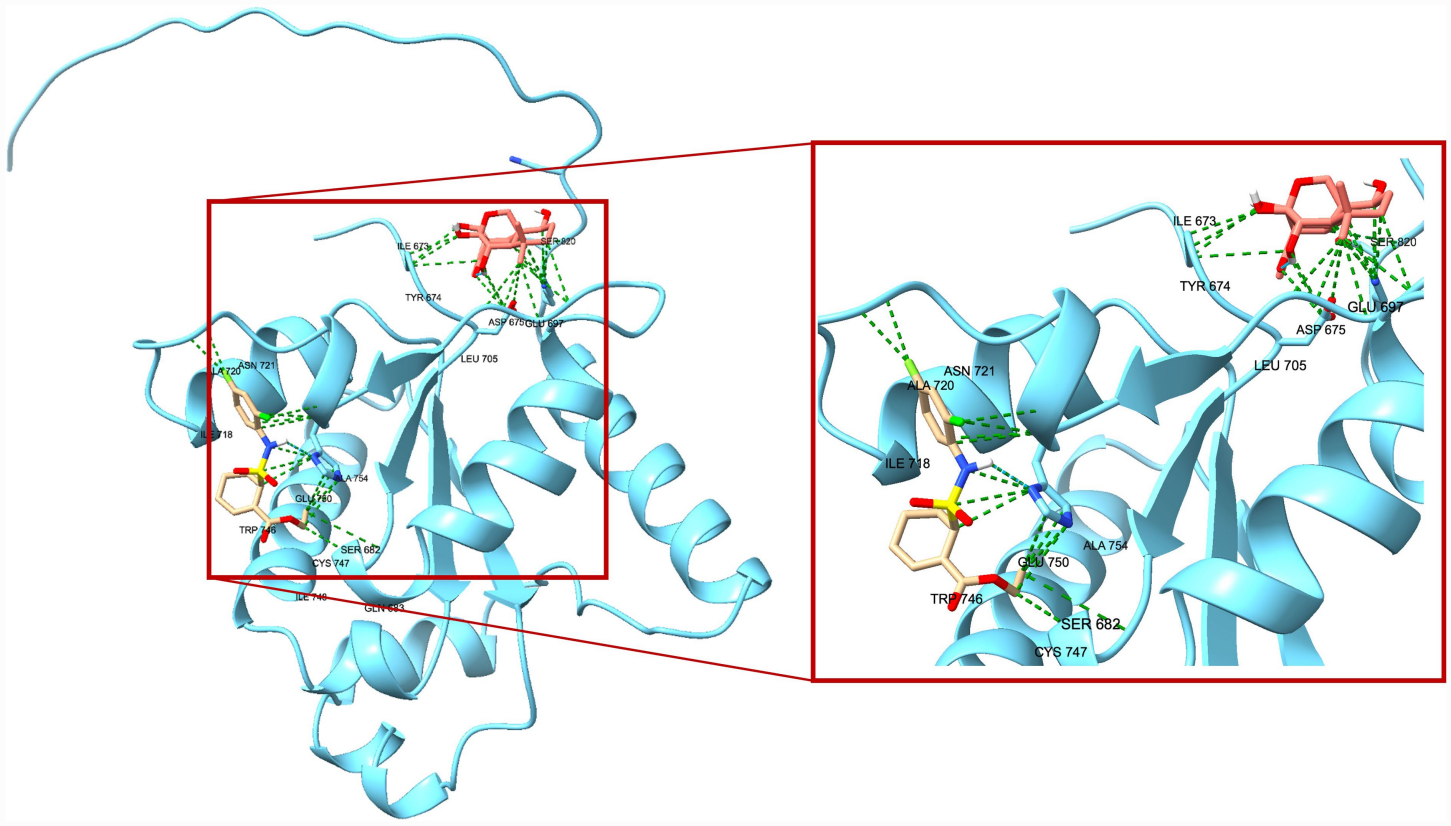
Comparative visualization of the binding modes of oridonin (pink) and TAK-242 (beige) within the TLR4-TIR domain. Both ligands are shown in stick representation, with the surrounding TLR4-TIR domain displayed in blue. Green dashed lines represent predicted van der Waals contacts, while cyan dashed lines indicate hydrogen-bond interactions stabilizing each ligand.

A total of 20 docking poses for each target (extracellular and intracellular domains) were generated. For each docking cluster, the Root Mean Square Deviation (RMSD) lower bound (L. B.) and upper bound (U. B.) were reported (Supplementary Tables S1–S4). These values reflect the minimum and maximum conformational deviation among poses in the same cluster, with lower RMSD indicating more consistent and reliable binding poses (Aier et al., 2016). For the extracellular TLR4-MD-2 complex, violin plots showing the distribution of predicted binding affinities (docking scores) of the ligands are presented (Figure 4), followed by representative visualizations of the most stable docking poses, including key interaction profiles such as hydrogen bonding and van der Waals forces (Figures 5–7). The same analytical and visualization strategy was applied to the intracellular TLR4-TIR domain with corresponding violin plots (Figure 8) and representative docking pose visualizations (Figures 9–11).

Docking scores are normally interpreted as moderate (< − 4.25 kcal/mol), good (< −5 kcal/mol), or significant (< −7 kcal/mol) (Yin et al., 2025). Docking scores for the extracellular TLR4-MD-2 complex were interpreted relative to TAK-242, a known pharmacological target of TLR4. It demonstrated binding energies ranging between −5.9 and −6.4 kcal/mol, consistent with prior reports of −5.8 to −6.9 kcal/mol for its interaction with the upper and lower TLR4 pockets (Tam et al., 2023). In contrast, oridonin exhibited substantially stronger docking energies (*p* < 0.0001, Figure 4), with its top-ranked poses ranging from −6.9 to −8.5 kcal/mol, falling within the “significant” affinity range. This finding suggests that oridonin may bind the TLR4-MD-2 complex with a higher predicted affinity than TAK-242. Both ligands localized to the same surface-exposed cavity at the upper region of the TLR4 ectodomain, distinct from the canonical MD-2 hydrophobic pocket that binds LPS (Figure 7). However, as the primary pharmacological mechanism of TAK-242 is reported to involve targeted interaction with the intracellular TIR domain, a separate docking analysis was performed for this region to evaluate whether oridonin could also modulate the receptor’s internal signaling machinery. Oridonin exhibited a significantly higher predicted binding affinity compared to TAK-242 (*p* < 0.0001; Figure 8). Specifically, the binding energies for TAK-242 in the TIR domain ranged from −5.18 to −5.88 kcal/mol, whereas oridonin showed a more favorable distribution of scores, ranging from −5.36 to −7.24 kcal/mol. The docking landscape within the intracellular TIR domain showed a distinct binding profile compared to that of the extracellular domain. Oridonin and TAK-242 localized to distinct regulatory sub-pockets within the intracellular signaling machinery (Figure 11).

## 4 Discussion

In this study, we demonstrate that oridonin exerts potent anti-inflammatory effects in LPS-stimulated EGCs by modulating the TLR4 signaling and autophagy pathway. A moderate (but sub-cytotoxic) inflammatory challenge with 10 μg/mL LPS caused significant glial activation without significant cell death, providing a model of subacute EGC inflammation. Oridonin, at low micromolar concentrations (1–5 μM), preserved EGC viability under these conditions and attenuated key markers of glial reactivity. Notably, oridonin significantly decreased the LPS-induced upregulation of S100B, an EGC activation marker, TLR4 itself, and the autophagy-related gene beclin-1, while also partially reducing the elevation of the autophagosome marker LC3. These findings suggest that oridonin effectively suppresses EGC inflammatory responses. By decreasing both inflammatory markers and autophagic changes, oridonin appears to maintain a more homeostatic glial phenotype in the endotoxin exposure.

LPS exposure is known to increase TLR4 expression in microglia and astrocytes, thereby enhancing their sensitivity to inflammatory stimuli and shifting them toward a proinflammatory phenotype (Rosciszewski et al., 2018). As a functional equivalent of brain astrocytes, EGCs also express TLR4 and respond to LPS by rapidly adopting a reactive, proinflammatory profile (Thomasi et al., 2023).

In line with these findings, our *in vitro* data showed that LPS stimulation significantly upregulated TLR4 and the glial activation marker S100B in EGCs. S100B is a Ca^2+^-binding protein abundantly expressed by EGCs, and excessive S100B release is associated with a proinflammatory glial phenotype that can disrupt enteric neuron function and gut motility (Turco et al., 2014). Oridonin effectively attenuated these LPS-induced increases, indicating that it modulates TLR4-associated inflammatory responses in peripheral glial populations and helps protect EGCs and preserve gastrointestinal function. This is particularly relevant in the context of chronic gut inflammation and neurodegenerative conditions. Indeed, TLR4-mediated EGC activation is thought to play a role in gut-brain axis dysfunction. TLR4 activation in both the CNS and the ENS is elevated in PD and likely contributes to early enteric pathology (Gorecki et al., 2021). Similar to our findings, multiple studies demonstrate that oridonin suppresses TLR4 signaling by downregulating TLR4 expression and inhibiting downstream pathways such as MyD88, NF-κB, and MAPK in the animal models of diabetic nephropathy (Li et al., 2018), endometritis (Zhou et al., 2019), acute liver injury (Shi et al., 2019), and acute lung injury (Zhao et al., 2017). However, it should be noted that canonical downstream signaling events of TLR4 activation, such as NF-κB p65 nuclear translocation or MAPK phosphorylation, were not directly assessed in the present study. Therefore, the observed effects of oridonin on TLR4 expression and inflammatory mediators in EGCs should be interpreted as modulation of TLR4-associated inflammatory responses rather than direct inhibition of the entire TLR4 signaling cascade. Mechanistically, oridonin’s anti-inflammatory profile in EGCs closely resembles that of the selective TLR4 antagonist TAK-242. In our experiments, TAK-242, a well-characterized small-molecule TLR4 inhibitor, produced similar downregulation of LPS-induced TLR4 and S100B, supporting the notion that both compounds mitigate EGC activation by targeting TLR4-related pathways. Notably, combining TAK-242 with oridonin did not further decrease mRNA levels, suggesting that oridonin’s regulatory effects may not be exclusively TLR4-dependent but rather involve downstream or parallel inflammatory pathways. Our molecular docking analysis revealed a dual-domain interaction profile for oridonin and TAK-242 across the TLR4 receptor. Within the extracellular TLR4-MD-2 complex, both ligands localized to an overlapping regulatory region, with oridonin displaying a stronger affinity for this regulatory site (binding energies −6.9 to −8.5 kcal/mol) compared with TAK-242 (−5.9 to −6.4 kcal/mol). We further investigated the intracellular TIR domain, the primary pharmacological target of TAK-242, to resolve the mechanism behind their observed synergy. They occupied distinct regulatory sub-pockets within the intracellular signaling machinery. Similar to binding scores of the extracellular domain, oridonin exhibited significantly higher binding energy in the TIR domain (−7.235 to −5.364 kcal/mol) than TAK-242 (−5.879 to −5.184 kcal/mol). This spatial divergence within the TIR domain suggests that oridonin and TAK-242 act as multi-site modulators rather than direct competitors for a single binding pocket. Based on these computational predictions, the observed pharmacological synergy could potentially arise from a complementary stabilization of different receptor domains, which may help maintain the TLR4 receptor in an inactive conformation.

Despite the increase in TLR4 mRNA expression, LPS stimulation produced only a modest rise in caspase-1 protein levels. Neither oridonin nor TAK-242 significantly reduced caspase-1 expression; however, co-administration of TAK-242 with LPS reduced caspase-1 activation in the combination groups (LPS + O1 and LPS + O2). Thus, our data indicate that oridonin alone did not produce a significant decrease compared with the LPS group, but when combined with the TLR4 antagonist TAK-242, it resulted in a marked reduction in cytokine levels. This finding suggests that the anti-inflammatory effect of oridonin may not be entirely TLR4-dependent but can be synergistically strengthened when TLR4 signaling is inhibited. Interestingly, in this study, inhibition of LPS-induced inflammatory signaling by TAK-242 was accompanied by increased IL-1β levels and decreased caspase-1 levels. This suggests that although TLR4 activation occurred at the transcriptional level, it may not have progressed to full inflammasome activation. Previous studies describe TLR4 signaling as a two-step process: the first step induces the synthesis of pro-IL-1β and pro-caspase-1, whereas the second requires inflammasome activation to convert these inactive precursors into their mature forms (Parzych et al., 2017). Moreover, mature IL-1β can be rapidly eliminated through proteasomal degradation or ubiquitination, which may result in stable IL-1β protein levels despite increased transcriptional activity of TLR4 (Vijayaraj et al., 2021). This phenomenon is well documented, showing that some cell types do not secrete IL-1β in response to LPS alone (Lu et al., 2012; Sanz and Di Virgilio, 2000). Although the rise in IL-1β despite TLR4 inhibition may appear unexpected at first glance, this pattern becomes meaningful when considering the biological function of caspase-1 and the mechanism of inflammasome activation. Caspase-1 is a central effector of the inflammatory response and proteolytically cleaves inactive precursors such as pro-IL-1β and pro-IL-18 into their mature forms (Antonopoulos et al., 2013). Therefore, once the inflammasome is activated, caspase-1 becomes enzymatically active, promotes the conversion of pro-IL-1β to mature IL-1β, and increases IL-1β release. In this context, the observed decrease in total caspase-1 levels together with elevated IL-1β may reflect the transition of caspase-1 into its active form. While the total amount of caspase-1 protein may decline, its enzymatic activity and IL-1β production may increase, consistent with enhanced IL-1β release following caspase-1 activation. Given that TAK-242 inhibits only TLR4 signaling, it is also possible that the second signal required for inflammasome activation proceeds through TLR4-independent pathways. As a result, the inflammasome complex may have remained active, allowing caspase-1-mediated IL-1β release to continue. Collectively, these findings suggest that although TLR4 serves as an important trigger of the inflammatory response, inflammasome and caspase-1 activation can proceed through alternative pathways independent of TLR4. This may explain why TAK-242 did not fully suppress inflammation and why IL-1β levels increased. The lower IL-1β output in oridonin-treated EGCs thus underscores oridonin’s dual mechanism: it not only inhibits TLR4/NF-κB-driven pro-IL1β induction, but also likely blocks the caspase-1-mediated processing of IL-1β. This dual suppression of both upstream and downstream portions of the inflammatory cascade may explain the superior anti-inflammatory efficacy of oridonin observed here. In the context of EGCs, such modulation of inflammasomes could be highly beneficial, as IL-1β is a potent cytokine that can disrupt enteric neural circuits and barrier function. By attenuating IL-1β release, oridonin might prevent a cascade of immune cell recruitment and secondary inflammation that would otherwise occur in the gut wall.

We also examined the expression of LC3 and beclin-1, two key markers of autophagy. Autophagy is a protective process through which cells remove damaged organelles and proteins, and it plays a critical role in maintaining homeostasis in neurodegenerative diseases. Impaired autophagy in PD has been proposed to contribute to α-synuclein accumulation and subsequent neuronal loss (Choi et al., 2022). LC3 and beclin-1 are fundamental proteins associated with autophagy. Beclin-1 plays a central role in initiating autophagosome formation, whereas LC3 is a marker of autophagosomal membranes and is commonly used to monitor autophagic activity (Hamurcu et al., 2018). Beclin-1 regulates the initiation of autophagy by interacting with other proteins, and its activity can influence LC3 lipidation and autophagosome formation (Sun et al., 2009). Although the effects of LPS exposure on autophagy are not entirely clear in the literature, it is known that, under conditions of severe inflammation, autophagic activity may be suppressed, insufficient, or increased (Ye et al., 2020; Guo et al., 2025; Xu et al., 2007). In our study, LPS markedly upregulated the mRNA levels of beclin-1 and LC3. While increased expression of these transcripts is often associated with autophagy induction, it is important to distinguish between functional autophagic flux and transcriptional stress responses. Induction of autophagy is a known response of glial cells to proinflammatory stimuli. Enteric glial autophagy can be triggered during inflammation and has been shown to facilitate antigen presentation and immune modulation (Deng et al., 2024). Intestinal tissue is highly enriched in TLR4 receptors and exhibits greater immune responsiveness to microbial products (Ortega-Cava et al., 2003). Therefore, in EGCs, excessive autophagy activation in response to LPS may emerge as a component of the inflammatory process. However, without assessing LC3-II protein conversion or p62/SQSTM1 degradation, it remains possible that the observed mRNA elevation reflects a compensatory response to an inflammatory challenge or a potential blockade in autophagic clearance rather than a complete activation of the pathway. Despite the lack of flux assays, the significant reduction of these transcripts by oridonin suggests that it mitigates the initial transcriptional stress response induced by LPS, which often accompanies chronic inflammation. This might preserve cellular energy balance and prevent autophagy-mediated cell damage, thereby contributing to the observed maintenance of cell viability.

This study has several limitations. First, the autophagic response was assessed primarily at the mRNA level, which reflects inflammatory and stress-related priming but does not directly assess functional autophagic flux or protein-level dynamics. Future studies incorporating protein-based analyses and flux modulation assays will be necessary to clarify these mechanisms. Second, while *in silico* docking suggested a favorable interaction between oridonin and TLR4, these findings remain predictive and were not supported by molecular dynamics simulations or direct biophysical binding assays. In addition, the putative TLR4 interaction sites identified by docking should be considered hypothesis-generating, as their functional regulatory roles in TLR4 signaling were not experimentally validated in the present study. Third, functional characterization of EGCs was limited to transcriptional analyses and ELISA-based cytokine measurements and did not include real-time functional readouts. Finally, the study relied on a single immortalized cell line. Although this provided a reproducible platform for mechanistic exploration, the absence of validation in primary cells, co-culture systems, or *in vivo* models limits direct extrapolation to complex physiological conditions.

In conclusion, our findings demonstrate that oridonin exerts a multifaceted protective effect on enteric glial cells exposed to LPS by concurrently dampening TLR4-driven inflammatory signaling and restraining aberrant autophagy induction. By suppressing TLR4 and S100B, and by upregulating beclin-1/LC3, oridonin effectively mitigates the inflammatory cascade. Given the critical role of enteric glial dysfunction in gut-brain axis disturbances and early PD pathology, oridonin may represent a promising candidate for targeting peripheral inflammation-driven neuroimmune mechanisms. Future studies exploring its effects in more complex gut-brain models and *in vivo* systems will be required to elucidate its therapeutic potential fully.

## Data Availability

The raw data supporting the conclusions of this article will be made available by the authors, without undue reservation.

## Footnotes

1
https://pubchem.ncbi.nlm.nih.gov/
