# Supplementary material for: Oridonin attenuates TLR4-driven inflammation and autophagy in LPS-stimulated enteric glial cells: an in vitro and in silico analysis
Source: Front Cell Neurosci. 2026 Jan 28;20:1748505. doi: 10.3389/fncel.2026.1748505 (PMC12890622; doi:10.3389/fncel.2026.1748505)
Supplement: Supplementary file 1 [file Table_1.docx]

**Supplementary Table 1.** RMSD and Binding Affinity Scores for Docking Models of Oridonin with TLR4-MD2 complex

| [**ID**](javascript:void(0);) | [**RMSD L.B.**](javascript:void(0);) | [**RMSD U.B.**](javascript:void(0);) | [**SCORE**](javascript:void(0);) |
| --- | --- | --- | --- |
| 1.1 | 0.000 | 0.000 | -8.487 |
| 1.2 | 7.473 | 11.005 | -8.446 |
| 1.3 | 9.078 | 11.463 | -8.353 |
| 1.4 | 1.639 | 2.644 | -7.814 |
| 1.5 | 4.826 | 8.064 | -7.744 |
| 1.6 | 3.915 | 6.517 | -7.550 |
| 1.7 | 3.736 | 6.306 | -7.453 |
| 1.8 | 8.334 | 11.125 | -7.395 |
| 1.9 | 9.423 | 12.128 | -7.391 |
| 1.10 | 7.551 | 9.257 | -7.387 |
| 1.11 | 8.980 | 11.573 | -7.365 |
| 1.12 | 4.250 | 6.609 | -7.364 |
| 1.13 | 16.271 | 18.909 | -7.128 |
| 1.14 | 16.795 | 19.759 | -7.107 |
| 1.15 | 7.643 | 10.915 | -7.061 |
| 1.16 | 5.193 | 7.527 | -7.058 |
| 1.17 | 16.843 | 19.604 | -7.040 |
| 1.18 | 16.140 | 18.857 | -7.027 |
| 1.19 | 8.585 | 13.187 | -7.011 |
| 1.20 | 8.451 | 11.146 | -6.915 |

**Supplementary Table 2.** RMSD and Binding Affinity Scores for Docking Models of TAK-242 with TLR4-MD2 complex

| [**ID**](javascript:void(0);) | [**RMSD L.B.**](javascript:void(0);) | [**RMSD U.B.**](javascript:void(0);) | [**SCORE**](javascript:void(0);) |
| --- | --- | --- | --- |
| 1.1 | 0.000 | 0.000 | -6.374 |
| 1.2 | 4.975 | 6.729 | -6.318 |
| 1.3 | 20.501 | 22.354 | -6.306 |
| 1.4 | 1.346 | 3.173 | -6.302 |
| 1.5 | 20.699 | 22.715 | -6.231 |
| 1.6 | 16.395 | 19.072 | -6.178 |
| 1.7 | 17.643 | 19.596 | -6.125 |
| 1.8 | 3.747 | 6.012 | -6.080 |
| 1.9 | 5.999 | 7.639 | -6.057 |
| 1.10 | 3.840 | 5.811 | -6.052 |
| 1.11 | 19.484 | 21.239 | -6.034 |
| 1.12 | 8.220 | 10.008 | -5.987 |
| 1.13 | 12.807 | 15.192 | -5.972 |
| 1.14 | 3.571 | 6.571 | -5.957 |
| 1.15 | 3.106 | 5.975 | -5.955 |
| 1.16 | 1.783 | 3.416 | -5.954 |
| 1.17 | 16.959 | 18.984 | -5.941 |
| 1.18 | 19.307 | 21.438 | -5.926 |
| 1.19 | 18.006 | 20.412 | -5.916 |
| 1.20 | 12.513 | 14.218 | -5.905 |

**Supplementary Table 3.** RMSD and Binding Affinity Scores for Docking Models of Oridonin with TLR4-TIR domain

| [**ID**](javascript:void(0);) | [**RMSD L.B.**](javascript:void(0);) | [**RMSD U.B.**](javascript:void(0);) | [**SCORE**](javascript:void(0);) |
| --- | --- | --- | --- |
| 1.1 | 0.000 | 0.000 | -7.235 |
| 1.2 | 1.512 | 5.750 | -6.911 |
| 1.3 | 2.085 | 4.123 | -6.591 |
| 1.4 | 2.097 | 5.873 | -6.317 |
| 1.5 | 2.717 | 4.906 | -6.173 |
| 1.6 | 22.884 | 24.764 | -6.150 |
| 1.7 | 3.276 | 7.144 | -6.136 |
| 1.8 | 1.744 | 5.446 | -6.103 |
| 1.9 | 2.429 | 4.537 | -6.089 |
| 1.10 | 2.874 | 4.827 | -6.048 |
| 1.11 | 22.278 | 24.568 | -6.030 |
| 1.12 | 22.925 | 24.773 | -5.997 |
| 1.13 | 23.203 | 25.452 | -5.910 |
| 1.14 | 2.172 | 5.402 | -5.880 |
| 1.15 | 2.436 | 4.557 | -5.820 |
| 1.16 | 3.054 | 5.722 | -5.772 |
| 1.17 | 23.034 | 25.030 | -5.705 |
| 1.18 | 23.261 | 24.576 | -5.621 |
| 1.19 | 17.819 | 20.835 | -5.589 |
| 1.20 | 23.865 | 25.917 | -5.364 |

**Supplementary Table 4.** RMSD and Binding Affinity Scores for Docking Models of TAK-242 with TLR4-TIR domain

| [**ID**](javascript:void(0);) | [**RMSD L.B.**](javascript:void(0);) | [**RMSD U.B.**](javascript:void(0);) | [**SCORE**](javascript:void(0);) |
| --- | --- | --- | --- |
| 1.1 | 0.000 | 0.000 | -5.879 |
| 1.2 | 1.589 | 1.722 | -5.688 |
| 1.3 | 25.343 | 26.433 | -5.654 |
| 1.4 | 24.968 | 26.172 | -5.638 |
| 1.5 | 24.901 | 26.107 | -5.528 |
| 1.6 | 3.587 | 5.996 | -5.501 |
| 1.7 | 25.402 | 26.633 | -5.498 |
| 1.8 | 24.685 | 25.883 | -5.463 |
| 1.9 | 25.204 | 26.553 | -5.447 |
| 1.10 | 3.647 | 5.943 | -5.440 |
| 1.11 | 23.214 | 24.587 | -5.438 |
| 1.12 | 3.687 | 5.421 | -5.430 |
| 1.13 | 2.381 | 2.981 | -5.368 |
| 1.14 | 24.327 | 25.941 | -5.356 |
| 1.15 | 24.438 | 25.800 | -5.341 |
| 1.16 | 2.097 | 4.470 | -5.326 |
| 1.17 | 24.240 | 25.514 | -5.284 |
| 1.18 | 24.105 | 25.403 | -5.280 |
| 1.19 | 24.695 | 26.066 | -5.201 |
| 1.20 | 23.354 | 24.566 | -5.184 |
